# Small mammal community in the largest urban forest in the Americas, southeastern Brazil

**DOI:** 10.1093/jmammal/gyag026

**Published:** 2026-04-28

**Authors:** Raquel Gonzalez Boullosa, Thiago dos Santos Cardoso, Leonardo Morgado, Mariana da Silva Batista, Sócrates Fraga da Costa Neto, Rosana Gentile

**Affiliations:** Laboratório de Biologia e Parasitologia de Mamíferos Silvestres Reservatórios, Instituto Oswaldo Cruz, Fundação Oswaldo Cruz, Av. Brasil 4365, Manguinhos, Rio de Janeiro, RJ 21040-360, Brazil; Programa de Pós-Graduação em Biodiversidade e Saúde, Instituto Oswaldo Cruz, Fundação Oswaldo Cruz, Av. Brasil, Manguinhos 4365, Rio de Janeiro, RJ 21040-360, Brazil; Laboratório de Biologia e Parasitologia de Mamíferos Silvestres Reservatórios, Instituto Oswaldo Cruz, Fundação Oswaldo Cruz, Av. Brasil 4365, Manguinhos, Rio de Janeiro, RJ 21040-360, Brazil; Laboratório de Hantaviroses e Rickettsioses, Instituto Oswaldo Cruz, Fundação Oswaldo Cruz, Av. Brasil 4365, Manguinhos, Rio de Janeiro, RJ 21040-360, Brazil; Laboratório de Biologia e Parasitologia de Mamíferos Silvestres Reservatórios, Instituto Oswaldo Cruz, Fundação Oswaldo Cruz, Av. Brasil 4365, Manguinhos, Rio de Janeiro, RJ 21040-360, Brazil; Fiocruz Mata Atlântica, Fundação Oswaldo Cruz, Jacarepaguá, Rio de Janeiro, RJ 22713-560, Brazil; Laboratório de Biologia e Parasitologia de Mamíferos Silvestres Reservatórios, Instituto Oswaldo Cruz, Fundação Oswaldo Cruz, Av. Brasil 4365, Manguinhos, Rio de Janeiro, RJ 21040-360, Brazil

**Keywords:** defaunation, *Didelphis aurita*, diversity, empty forest, mesopredator, urban areas, areas urbanas, defaunação, *Didelphis aurita*, diversidade, floresta vazia, mesopredador

## Abstract

Human activities directly influence the diversity, abundance and distribution of species. The loss of species in forested areas—called the defaunation process—is an outcome of anthropogenic processes, especially in forest remnants close to urban and rural areas. The aim of this study was to evaluate small mammal diversity in a forest–urban interface area at the largest urban forest in the Americas, in the city of Rio de Janeiro, Brazil. Seven small mammal species were captured: the marsupials *Didelphis aurita, Marmosa paraguayana*, *Metachirus myosuros*, and *Monodelphis americana*; and the rodents *Akodon cursor*, *Oligoryzomys nigripes*, and *Trinomys dimidiatus*. The common opossum *D. aurita* was dominant in the community. Beta diversity indices revealed little variation among types of environments and over time. Species richness of the small mammal community in the study area was lower than that reported in the literature. The study area contained a fixed set of core species in the community over time and among environments (peridomicile, disturbed forest, and preserved forest), with the presence of less abundant species in some periods and environments. Small mammal species richness observed was lower than in preserved areas, when compared with other studies. Historical, ecological, and anthropogenic characteristics of the area may have contributed to its low diversity.

## Teaser

A small mammal community was studied in a peri-urban Atlantic Forest area. The results revealed little variation among types of environments and over time, and the small mammal species richness was lower than in preserved areas.

## Resumo

As atividades humanas influenciam diretamente a diversidade, abundância e distribuição das espécies. A perda de espécies em áreas florestais, chamada de processo de defaunação, é resultado de processos antropogênicos, especialmente em remanescentes florestais próximos a áreas urbanas e rurais. O objetivo deste estudo foi avaliar a diversidade de pequenos mamíferos em uma área de interface floresta-urbano na maior floresta urbana das Américas na cidade do Rio de Janeiro, Brasil. Sete espécies de pequenos mamíferos foram capturadas, os marsupiais *Didelphis aurita*, *Marmosa paraguayana*, *Metachirus myosuros* e *Monodelphis americana*, e os roedores *Akodon cursor*, *Oligoryzomys nigripes* e *Trinomys dimidiatus*. O gambá comum *D. aurita* foi dominante na comunidade. Os índices de beta-diversidade revelaram pouca variação entre os tipos de ambientes e ao longo do tempo. A riqueza de espécies da comunidade de pequenos mamíferos na área de estudo foi menor do que as relatadas na literatura. A área de estudo apresentou um conjunto fixo de espécies centrais na comunidade ao longo do tempo e entre os ambientes (peridomicílio, floresta perturbada e floresta preservada), com a presença de espécies menos abundantes em alguns períodos e ambientes. A riqueza de espécies de pequenos mamíferos observada foi menor do que em áreas preservadas, quando comparada a outros estudos. As características históricas, ecológicas e antropogênicas da área podem ter contribuído para sua baixa diversidade.

Urbanization and the expansion of human socioeconomic activities in forested areas reduce, alter, and fragment natural habitats—influencing the diversity, abundance, and distribution of species (Pires et al. 2006; Gomes et al. 2011). The loss of vertebrate fauna (defaunation) in forested areas led to what Redford (1992) called empty forest, as defaunation can occur with or without habitat loss or habitat fragmentation (Bogoni et al. 2023). Top predator species are the most affected by anthropic factors (Barros et al. 2021), and for this reason, a decline in top predator species is commonly associated with an increase in the abundance of smaller predator species—a process called mesopredator release (Prugh et al. 2009). In forest areas close to urban environments, some species of small mammals can be favored not only by the absence of top predators but also by the food supply originating from domestic waste (Fardel and Dickman 2023). As a result, the abundance of generalist or opportunistic wild species and synanthropic species increases (Gentile et al. 2018; Simioni et al. 2022), indicating that some species are more sensitive to anthropogenic changes than are others (Gatto-Almeida et al. 2023).

The Atlantic Forest is part of the tropical forest biome, the second largest tropical rainforest of the American continent after the Amazon Forest (Marques et al. 2021), and a world biodiversity hotspot (Mittermeier et al. 1998; Lima et al. 2020). This biome has undergone fragmentation processes over time since European colonization. According to Vancine et al. (2024), it represents approximately 22.9% of forest vegetation cover and is considered one of the most diverse and threatened biomes in the world. The Atlantic Forest houses approximately 384 mammal species (Figueiredo et al. 2021), including approximately 32 species of marsupials (Delciellos et al. 2022) and 108 species of rodents (Graipel et al. 2017). Approximately 98% of the mammal biomass of the Atlantic Forest is estimated to have declined in areas of intensive hunting (Galetti et al. 2017). In areas of the Atlantic Forest where top predators are locally extinguished, the abundance of mesopredator species such as common opossums, foxes, coatis, and raccoons is expected to increase (Gentile et al. 2018; Pires and Galetti 2023).

Small non-volant mammals play important roles in terrestrial ecosystems as seed dispersers, arthropod predators, population regulators (Larson and Sander 2025), and reservoirs of zoonoses (Bonvicino et al. 2014). Most surveys of small non-volant mammals in the Atlantic Forest have been carried out in preserved forest reserves in conservation units such as national or state parks (Geise et al. 2004; Viveiros De Castro and Fernandes 2004; Cunha and Rajão 2007; Modesto et al. 2008a; Cronemberger et al. 2019; Delciellos et al. 2023), among which most reported high species richness, ranging from 10 to 30 species. Some studies were carried out in rural areas, private forest fragments, or small reserves surrounded by rural properties (D’Andrea et al. 2007; Vaz et al. 2007; Modesto et al. 2008b; Pessôa et al. 2009; Gonçalves et al. 2016), which reported from 4 to 17 species associated with local habitat characteristics. Other studies have been conducted in forested areas close to urban centers in peri-urban environments (Freitas et al. 2006; Oliveira et al. 2007; 2012; Guerra and Leite 2017; Gentile et al. 2018; Travassos et al. 2018; Monticelli et al. 2021), most of which reported low species richness.

Among the remaining Atlantic Forest fragments in the state of Rio de Janeiro, Pedra Branca State Park (PEPB hereafter) stands out in the metropolitan region of the city of Rio de Janeiro, as it is the largest urban forest of the Americas, covering 12,000 ha. This forest remnant comprises areas above 100 m in altitude. The FIOCRUZ Mata Atlântica Biological Station (EFMA hereafter) is located within the PEPB and adjacent areas encompassing 430 ha at the east slope of the Pedra Branca massif, where 262 ha overlap with the PEPB (Moratelli et al. 2023). This region was occupied by sugarcane plantations and coffee mills in the 18th century and later began to have centers of human occupation and a broad urbanization process around it (Corrêa 2017). From 2003 onward, the area was incorporated into the Oswaldo Cruz Foundation (FIOCRUZ), where projects of environmental preservation, reforestation, fauna monitoring, and pathogen diagnosis were developed based on transdisciplinary approaches according to the concept of one health (Moratelli et al. 2023).

Since 2012, a project involving taxonomic surveys, ecology, genetics, and parasitology of small mammals (rodents and marsupials) has been developed at EFMA (Gentile et al. 2018; Luz et al. 2018; Costa-Neto et al. 2019; Valença-Barbosa et al. 2019; Berbigier et al. 2021; Boullosa et al. 2021; Gentile et al. 2023). This project has had several stages meeting; the objectives of a wide group of interdisciplinary collaborators, involving aspects of small mammal biodiversity and diagnoses of their pathogens and parasites, with emphasis on etiological agents related to zoonoses associated with small non-volant mammal species. Considering this context, the aim of this study was to compare the species richness and abundance of small mammals at EFMA between 3 kinds of environments along a gradient of anthropogenic action (peridomicile, disturbed forest, and preserved forest) and over time from 2012 to 2024, considering 3 periods of study using alpha (within localities) and beta (between localities) diversity indices. We also compared the species composition and species richness of the study area with those of other surveys of small mammals carried out in the Atlantic Forest of Southeast Brazil. We expected to find a reduction in species richness in our study area compared with preserved areas of the Atlantic Forest of southeastern Brazil because it is an area of forest–urban interface.

## Methods

### Study area

**Fig. 1:**
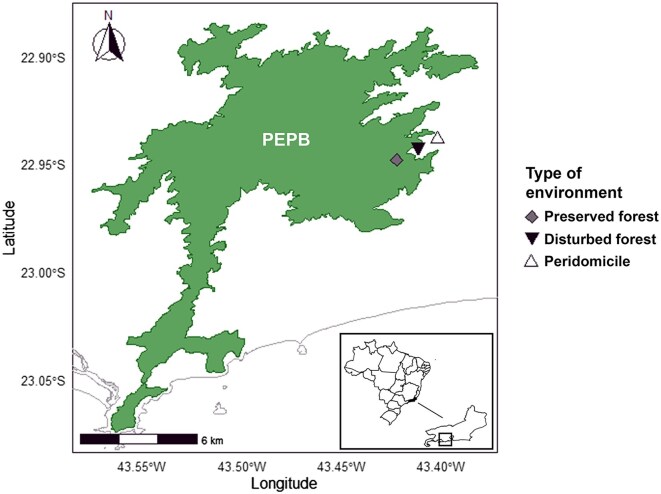
Map of the study area indicating sampling localities at the Fiocruz Atlantic Forest Biological Station, State of Rio de Janeiro, Brazil.

The study was carried out at EFMA, including areas on the edge of the forest that encompass the PEPB in the city of Rio de Janeiro. Thus, the study was conducted at an interface area of forest and urban environments in the west zone of Rio de Janeiro city (Fig. 1). Climate of the region is humid tropical with no dry season, hot rainy summers, and mild winters (Ayoade 1986). The study was carried out in 3 different types of environments: (i) peridomicile (PA-22°56′18.8″S 43°24′5.8″W; PB-22°56′12″S 43°24′0.4″W; PC-22°56′9.9″S 43°24′1.2″W), an area close to human habitations with low canopy heights varying from 0 to 10 m, predominance of shrubs and short trees, presence of some flooded areas, and slopes varying from flat to moderately steep; ii) disturbed forest (TA- 22°56′28.6″S 43° 24′33.2″W; TB- 22°56′27.1″S 43°24′35.5″W and TC- 22°56′29.6″S 43°24′33.5″W), which is a dense ombrophilous forest area in the process of regeneration and reforestation that is considered a transition area between the other 2 environments with canopy heights varying from 6 to 19 m and flat slopes; and (iii) preserved forest (FA-22°56′39.9″S 43°25′00.3″W; FB-22°56′42.4″S 43°24′58.5″W and FC-22°56′40.4″S 43°24′58.6″W), which is an area with dense rainforest vegetation with canopy heights varying from 10 to 25 m and steep relief, more than 1 km away from the nearest edge of the forest fragment that forms the PEPB.

### Small mammal sampling

The study was divided into 3 periods. The first period included a survey in which the animals were removed and euthanized, except for lactating females, for species identification and collaborative studies of pathogen diagnoses. These identifications were performed by the team from the National Reference Service for Taxonomy and Diagnosis of Wild Leishmaniasis Reservoirs of Instituto Oswaldo Cruz. Voucher specimens were deposited in the Mammal Collection of Museu Nacional do Rio de Janeiro. In this first phase, captures were carried out in 6 transects of 20 trapping stations during 6 sampling events from July 2012 to November 2014. A detailed description of the study area of this preliminary phase and the methodology of species identification is reported in Gentile et al. (2018). From September 2018 to December 2019, a second phase of a mark-recapture study was carried out on 9 transects of 10 trapping stations in the same types of environments and areas of the first phase during 6 sampling events (Boullosa 2022), which were interrupted because of the pandemic. In March 2023, we continued the mark-recapture study in 5 transects of 15 trapping stations in the same areas. This third phase included 4 sampling events until September 2024. In the first phase of the study, each sampling event had 5 consecutive trapping-nights. In the other phases, the sampling events had 4 consecutive trapping-nights. During these last 2 phases, species that were not captured in the first phase were removed for species identification using the same methods described in Gentile et al. (2018).

At each trapping station, Sherman (7.62 cm × 9.53 cm × 30.48 cm) and Tomahawk (40.64 cm × 12. 70 cm × 12.70 cm) live traps were placed on the ground. The traps were baited with a mixture of banana, bacon, peanut butter, and oats and checked daily during each collection period. Animals were captured under authorization of the Brazilian Government’s Chico Mendes Institute for Biodiversity and Conservation (ICMBIO, license number 13373) and the Environmental Institute of Rio de Janeiro state (INEA, license number 020/2011). All procedures followed the guidelines for the capture, handling, and care of animals of the Ethical Committee on Animal Use of the Oswaldo Cruz Foundation (CEUA, license numbers LW-39/14). Biosafety procedures and personal safety equipment were used during all procedures involving animal handling and biological sampling. The captured animals during the mark-recapture phases were individually marked with numbered ear tags (National Band & Tag Company), weighed, and measured. In cases of doubt regarding identification during the mark-recapture phases, animals were removed for species confirmation.

### Data analyses

Capture success was calculated as the ratio between the number of captures and the total effort used in traps per night for each period. Species richness was considered to be the number of species captured in each type of environment and each period of study. The abundance of small mammals was considered to be the number of individuals captured per species in each type of environment and each period of study, disregarding recaptures.

Rarefaction and extrapolation curves of species richness and diversity were generated from abundance data for each period of study and for each type of environment. The rarefaction and extrapolation curves were based on the Hill numbers (*q* = 0, representing species richness; *q* = 1, representing Shannon diversity; and *q* = 2, representing Simpson diversity) according to Chao et al. (2014). Rarefaction curves were constructed to compare diversity between communities (types of environments and sampling periods), whereas extrapolation curves were constructed to assess the estimated diversity and compare it to the observed diversity of each community (Chao et al. 2014).

We calculated the multiple-site beta diversity (*β*) and decomposed it into components of balanced variation (beta.BRAY. BAL)—analogous to turnover—and abundance gradients (beta.BRAY. GRA)—analogous to nestedness—for abundance data (Baselga 2017). The balanced variation component has the maximum value (1) when no species are present at more than 1 site, whereas the abundance-gradient component has the minimum value (0) when there are no species in common (Baselga 2017). These analyses were performed to investigate whether diversity was driven by the replacement or loss of species and individuals in the community. This analysis was carried out: (i) among the three kinds of environments considering all periods; and (ii) among the study periods (2012 to 2014, 2018 to 2019, and 2023 to 2024). The beta diversity indices and their components were determined based on the Bray–Curtis distance because we considered abundance data.

We compared the total small mammal species richness and species composition of the study area (all periods and all environments) with those of other small mammal surveys conducted in areas of dense ombrophilous forest in the Atlantic Forest in southeastern Brazil. For this purpose, we selected 10 published studies (Supplementary Data SD1) that were carried out in forested areas of the Atlantic Forest either in preserved localities (6 preserved areas) or in forests close to urban centers (4 peri-urban areas). We filtered published articles to meet our criteria detailed below using the database of Bovendorp et al. (2017), and searching in Web of Science, Scielo, and Google Scholar databases using the keywords “small mammals,” “Atlantic Forest,” “Brazil,” and “urban.” We included published studies that used live traps in which it was possible to filter only captures on the ground made in that kind of trap. We did not include studies carried out in mixed ombrophilous forests, southern and northeast Atlantic Forest, high altitude grasslands, rural areas, restingas, deciduous or semideciduous forests, and studies that were not surveys of small mammals. Thus, we selected studies that met our criteria to make them comparable with the regional species pool. The species nomenclature cited in the articles was corrected and updated according to Gardner (2008), Faria et al. (2019), and Patton et al. (2015). Only wild small mammal species captured in live traps placed on the ground were included in this analysis. For species composition comparisons, a nonmetric multidimensional scaling (NMDS) analysis was carried out based on a matrix of the presence and absence of species in each community (sampled studies) using Jaccard distance to investigate the dissimilarity in species composition between studies. The goodness of fit was assessed using the stress measure, which indicates the proportion of variation in original distances in relation to distances predicted by the NMDS (Legendre and Legendre 2012). In this case, the stress value varies from 20% (poor) to 0% (perfect), being considered fair to good between 10% and 5%, and excellent below 2.5% (Kruskal 1964). For species richness comparisons, we used the number of species detected in each study. In addition, a Permutation Multivariate Analysis of Variance (PERMANOVA) was performed to assess the significance of species composition differences between studies conducted in preserved and peri-urban areas. The significance level considered was set at 0.05.

The beta diversity indices and their components were calculated using the “betapart” package (Baselga et al. 2021), the rarefaction/extrapolation curves and estimated richness values were calculated using the “iNEXT” package (Chao et al. 2014), and the Jaccard indices, NMDS analysis, and PERMANOVA were performed using the “vegan” package (Oksanen 2024), all of which were in R software version 4.4.2 (R core team 2024).

## Results

Ninety-five animals were captured from 2012 to 2014, and 85 animals were captured from 2018 to 2019 of which only 3 were recaptured, and 45 were captured from 2023 to 2024. The sampling effort was 11,200 trap-nights, and the capture success rates were 0.84% from 2012 to 2014, 4,752 trap-nights and 1.79% from 2018 to 2019, and 2,560 trap-nights and 1.76% from 2023 to 2024. Seven species of small wild mammals were captured, considering the 3 types of environments and the 3 periods of study (Table 1). Among these, 4 species were of the order Didelphimorphia—*Didelphis aurita* (Wied-Neuwied, 1826), *Marmosa paraguayana* (Tate, 1931), *Metachirus myosuros* (Temmink, 1824), and *Monodelphis americana* (Müller, 1776); and 3 species were of the order Rodentia—*Akodon cursor* (Winge, 1887), *Oligoryzomys nigripes* (Olfers, 1818), and *Trinomys dimidiatus* (Günther, 1877).

**Table 1:** Abundance of small mammals captured in each type of environment (peridomicile, disturbed forest, and preserved forest) and period of study (2012 to 2014, 2018 to 2019, and 2023 to 2024) at FIOCRUZ Atlantic Forest Biological Station (EFMA), state of Rio de Janeiro, Brazil.

	2012 to 2014			2018 to 2019			2023 to 2024		
Species	Peridomicile	Disturbed forest	Preserved forest	Peridomicile	Disturbed forest	Preserved forest	Peridomicile	Disturbed forest	Preserved forest
**Family Didelphidae**									
*** Didelphis aurita***	39	28	9	42	20	9	18	12	2
*** Marmosa paraguayana***	1	0	3	4	1	0	3	0	0
*** Metachirus myosuros***	0	1	0	0	0	0	0	0	2
*** Monodelphis americana***	0	0	2	0	0	2	1	1	0
**Family Cricetidae**									
*** Akodon cursor***	7	0	0	0	0	0	0	0	0
*** Oligoryzomys nigripes***	3	1	1	3	1	2	0	2	4
**Family Echimydae**									
*** Trinomys dimidiatus***	0	0	0	0	1	0	0	0	0
**Species richness**	4	3	4	3	4	3	3	3	3
**Total abundance**	50	30	15	49	23	13	22	15	8

**Fig. 2:**
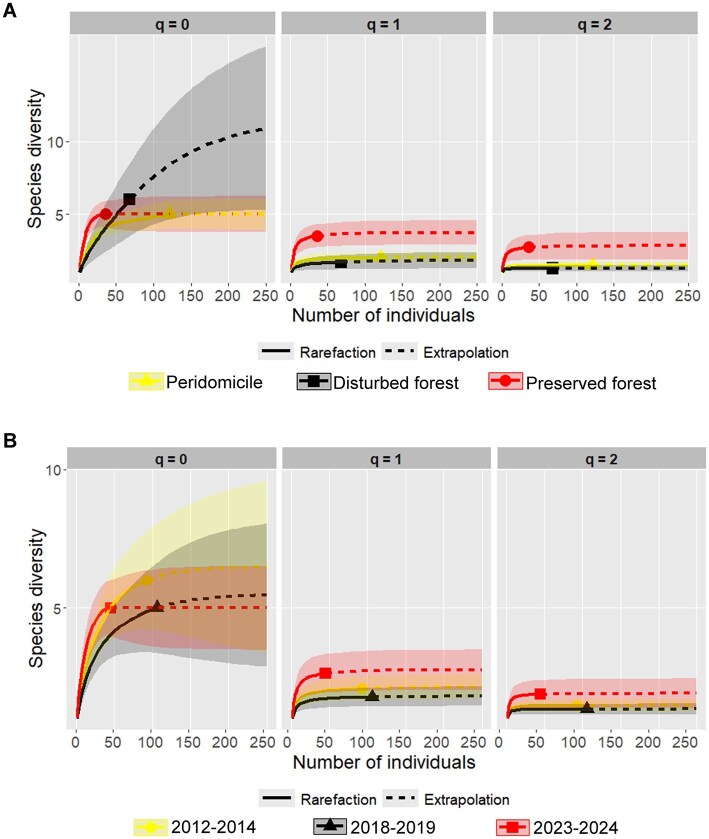
Plots of rarefaction and extrapolation curves of species richness and diversity from abundance data for (A) type of environment (peridomicile, disturbed forest, and preserved forest), and for (B) each period of study (2012 to 2014, 2018 to 2019, and 2023 to 2024). The rarefaction and extrapolation curves were based on the Hill numbers (*q* = 0, representing species richness; *q* = 1, representing Shannon diversity; and *q* = 2, representing Simpson diversity), according to Chao et al. (2014).

The most abundant species was the opossum *D. aurita*, which was found in all environments and periods of study, with greater abundance in the peridomicile (Table 1). The observed species richness ranged from 5 to 6 species per environment (Table 1). The estimated species richness (*q* = 0, extrapolation curve) was greater in the disturbed forest (11.9 ± 3.8), followed by the preserved forest (5 ± 0.6), and peridomicile (5 ± 0.5; Fig. 2A). In relation to the diversity indices (*q* = 1 and *q* = 2), the greatest values were obtained for the preserved forest, either in the rarefaction or in the extrapolation curves (Fig. 2A).

Comparing the 3 periods of study, the estimated species richness (*q* = 0, extrapolation curve) was greater from 2012 to 2014 (6.5 ± 1.9) than from 2018 to 2019 (5.5 ± 1.3) and 2023 to 2024 (5 ± 0.8; Fig. 2B). However, the observed and extrapolated diversities (*q* = 1 and *q* = 2) were greater from 2023 to 2024 (Fig. 2B).

A low beta diversity index was observed among the 3 environments, and an intermediate beta diversity index was observed among periods (2012 to 2014, 2018 to 2019, and 2023 to 2024; Table 2). For both environments and periods, we recorded greater species loss than species replacement (Table 2).

**Table 2:** Beta diversity indices, turnover, and nestedness components of small mammals among the types of environments (peridomicile, disturbed forest, and preserved forest) and the periods of study (2012 to 2014, 2018 to 2019, and 2023 to 2024) at FIOCRUZ Atlantic Forest Biological Station (EFMA), state of Rio de Janeiro, Brazil.

Comparisons	Beta Diversity (beta.BRAY)	Turnover (beta.BRAY.BAL)	Loss (beta.BRAY.GRA)
**(1) Types of environments**	0.51	0.15	0.36
**(2) Periods of study**	0.37	0.10	0.27

**Fig. 3:**
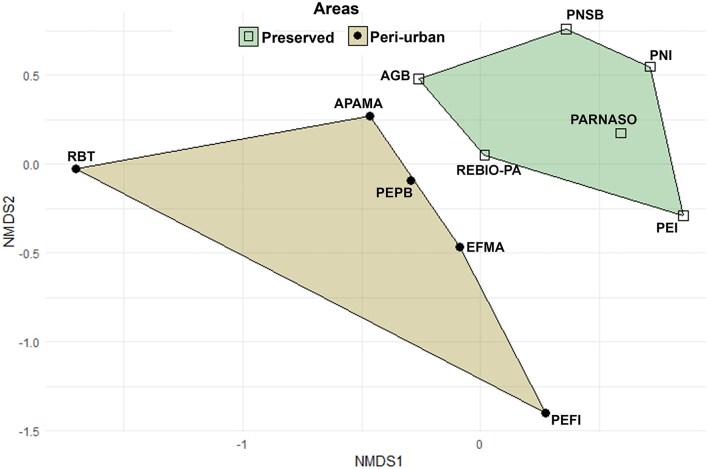
Similarity in species composition of small mammals among studies carried out in preserved and peri-urban forests in Atlantic Forest, Brazil, available in the literature and studies carried out at the Fiocruz Atlantic Forest Biological Station, using nonmetric multidimensional scaling (NMDS) analysis. The black circles represent studies conducted in peri-urban forests, and the open squares represent studies conducted in preserved forests. Each locality corresponds to a different study according to Supplementary Data SD1. We included only studies and data that were carried out in dense ombrophilous forests in Southeast Brazil that used live traps in which it was possible to filter only captures on the ground made in that kind of trap.

The NMDS result (stress = 0.13; Fig. 3) indicated that the greatest dissimilarity in species composition occurred between PEFI (Monticelli et al. 2021; peri-urban) and APAMA (peri-urban; Guerra and Leite 2017), which had species richness values equal to 5 and 10, respectively (Supplementary Data SD1). In turn, the greatest similarity in species composition occurred between PEPB (Oliveira et al. 2012; 8) and EFMA (present study; 7), both in peri-urban forest areas of the same reserve (Supplementary Data SD1). PERMANOVA revealed significant differences in species composition between studies conducted in preserved and peri-urban areas, which formed 2 separate polygons according to each category (*F *= 1.80, DF = 9, *R*^2^ = 0.17, *P* = 0.04; Fig. 3).

## Discussion

The most important finding of the present study was that there was a fixed set of core species in the community with little variation among types of environments and over time, according to the beta-diversity analyses. In addition, the small mammal species richness was lower than in preserved areas compared with other studies. Among the 7 species recorded in the study area, only *D. aurita*, *M. paraguayana*, *M. americana*, and *O. nigripes* were recorded in the 3 periods. The marsupial *M. myosuros* was recorded only in the first and last sampling periods, whereas the rodent *A. cursor* was recorded only in the first sampling period, and the rodent *T. dimidiatus* was recorded only in the second period. In addition, the marsupial *Gracilinanus microtarsus* was recorded in this area in 2001 (Gentile et al. 2018) during a sampling that was carried out for the development of a key plan for the management and conservation of the campus just before this area was incorporated into FIOCRUZ in 2003, but was not subsequently registered. The opossum *D. aurita* was the dominant species, confirming its ability to adapt to different types of habitats, particularly disturbed environments close to human habitations (Graipel and Santos-Filho 2006). In peridomicile areas there may be an increase in food availability, which favors generalist species such as the opossum (Gentile et al. 2018) which feeds on various items found close to human habitations (Santori et al. 2012). In wild/urban or wild/rural interface areas, opossum population sizes are larger than those in preserved forest areas (Gentile et al. 2004; D’Andrea et al. 2007; Kajin et al. 2008). Compared with a study carried out in the PEPB, Oliveira et al. (2012) recorded 9 species of small wild mammals between 2005 and 2007, 3 species of rodents: *A. cursor*, *O. nigripes*, *Guerlinguetus brasiliensis*, and 6 species of marsupials: *D. aurita*, *M. incanus*, *M. paraguayanus*, *M. myosuros* (formerly *M. nudicaudatus*), *M. americana*, and *P. quica* (formerly *P. frenatus*). The species *M. incanus* and *P. quica* had not been registered at the EFMA limits until now.

The beta diversity results indicated that there was a greater difference in species composition and abundance in space (among environments) than over time at EFMA, with greater species loss than replacement along the environmental gradient. Despite this, beta diversity was not high across environments or periods. The species loss recorded can be attributed to seasonal fluctuations and differences between environments, where only generalist species had high abundance throughout the study in all the environments. In this way, species composition and abundance varied along the spatial and temporal gradient, with only *D. aurita* present in the 3 environments and sampling periods and with high abundance. In fragmented or disturbed forests, the population sizes of some species of wild small mammals may increase due to their generalist characteristics or due to the local extinction of their predators (Delciellos et al. 2016; Pires and Galetti 2023).

Both the rarefaction and extrapolation curves and the beta diversity indices indicated greater differences between types of environments than over time in the study area. The rarefaction and extrapolation curves for both richness and diversity showed considerable overlap between periods, considering the confidence intervals, which indicates little temporal variation in the richness and diversity of small mammal species in the study area. However, comparisons across environments indicated greater diversity in the preserved forest than in the other environments, which was less clear in relation to richness due to the overlap of the curves. However, as mentioned above, beta diversity was low both in relation to periods and in relation to environments, with greater species loss than species turnover in space and time. As noted by Hobbs et al. (2018), the movement of species is influenced by complex interactions of environmental changes, resource availability, competition, human actions, and species characteristics. Thus, the disturbed forest environment allows individuals to move between the preserved forest and peridomicile, increasing ecological connectivity between these environments. In fact, all mammal species recorded in the preserved forest and peridomicile, with the exception of *A. cursor*, were found in the disturbed forest. Preserved forests may have more stable environmental conditions (such as temperature, humidity, and soil quality), which are favorable for the establishment and survival of a wide range of species (Hautier et al. 2015) and may have contributed to greater estimated diversity in the forest environment.

The results indicate that the study area seems to have a fixed set of species, with variations in the occurrence of species over time possibly resulting from variations in their abundances. The low species diversity observed may be the result of a set of factors that occur in the study area. First, the history of marked environmental degradation promoted changes in land use and in the phytosociological profile over time (Corrêa 2017). Second, there is an absence of top predator species (Veríssimo et al. 2023). We suggest that this fact may have led to a reduction in species diversity through a cascade effect in the food web which may have contributed to an increase in the abundance of mesopredator species (*D. aurita*; Pires and Galetti 2023), generalist species (*D. aurita* and *M. paraguayana*; Cáceres et al. 2022), and species with opportunistic reproduction models (*O. nigripes*; Cerqueira 2005). Veríssimo et al. (2023) also reported the occurrence of other species of mesopredators in the study area in addition to the opossum, including *Cerdocyon thous, Leopardus guttulus, Procyon cancrivorus*, and *Nasua nasua*. According to Prugh et al. (2009), mesopredators, which become the dominant species, are common in fragmented environments. Third, the proximity of human habitations may have created favorable conditions for the occurrence of certain generalist or opportunistic species in terms of habitat use due to the abundance of food resources (Gentile et al. 2018). In addition, there are many domestic dogs (*Canis familiaris*) in the study area (Veríssimo et al. 2023) that, although domiciled, move freely in the forest and may threaten the native fauna, whether by predation, competition, or disease transmission.

The NMDS analysis clearly revealed a separation between preserved and peri-urban studies in relation to species presence, as expected, which was corroborated by the PERMANOVA result. In addition, there were more dissimilarities among the peri-urban studies than among the preserved studies, suggesting greater environmental heterogeneity among the peri-urban localities than among the preserved localities. These factors may contribute to greater turnover of species according to the characteristics of each area. Preserved areas may have better environmental quality for small mammal species, favoring specialist species in addition to the species present in the peri-urban areas. The species composition of this study was more similar to that of the PEPB, as expected, because it is located in the same urban reserve where the EFMA is inserted. This result can be attributed to the presence of the species *D. aurita*, *M. paraguayana*, *M. americana*, *M. myosurus*, *O. nigripes*, and *A. cursor* in these areas. The species richness in our study area was lower than that reported in studies carried out in the 6 preserved areas: Cronemberger et al. (2019) = 30; Cunha and Rajão (2007) = 10; Delciellos et al. (2023) = 23; Geise et al. (2004) = 28; Vieira and Monteiro-Filho (2003) = 18; and Viveiros De Castro and Fernandes (2004) = 12. The studies carried out close to urban areas included in the comparison also reported low species richness: Guerra and Leite (2017) = 9; Monticelli et al. (2021) = 5; Oliveira et al. (2012) = 8; and Travassos et al. (2018) = 6.

This study revealed that human activities in this forest edge area exerted strong pressure on the environment, resulting in a low diversity of small mammals, despite being temporally stable as indicated by the low beta diversity over time. The absence of top species may have increased the abundance of generalist, opportunist, and mesopredator species, resulting in a reduction in species diversity and changes in the food web. Given the insertion of this forest fragment in a densely populated urban area, it is necessary to highlight that these factors affect the trophic structure of the community and, consequently, ecosystem functions. Although taxonomic diversity may be correlated with other diversity dimensions, the use of multiple dimensions is important for characterizing various aspects of biodiversity (de la Sancha et al. 2020). A promising approach for future studies is the analysis of functional diversity of the mammalian community and the effect of the environment on its functional homogenization.

## Supplementary Material

gyag026_Supplementary_Data

## Data Availability

Supporting data are available in Table 1 and in Supplementary Data SD1.

## Supplementary data

**Supplementary Data SD1**. Table S1. Surveys of small mammal species captured on the ground using live-traps, which were carried out in preserved and peri-urban dense ombrophilous forests in Southeast Atlantic Forest.
