# Supplementary material for: Small mammal community in the largest urban forest in the Americas, southeastern Brazil
Source: J Mammal. 2026 Apr 28;107(3):627–36. doi: 10.1093/jmammal/gyag026 (PMC13271762; doi:10.1093/jmammal/gyag026)
Supplement: gyag026_Supplementary_Data [file gyag026_supplementary_data.docx]

**Supplementary Data SD1**. Table S1. Surveys of small mammal species captured on the ground using live-traps, which were carried out in preserved and peri-urban dense ombrophilous forests in Southeast Atlantic Forest.

| **Regions of the state of Rio de Janeiro** | **Code** | **Locality** | **Species Richness** | **Reference** |
| --- | --- | --- | --- | --- |
| Periurban | EFMA | Estação Biológica Fiocruz Mata Atlântica – Rio de Janeiro | 7 | Present sturdy |
| Periurban | PEPB | Parque Estadual da Pedra Branca – Rio de Janeiro | 8 | Oliveira et al. 2012 |
| Periurban | APAMA | APA Mestre Álvaro - Vitória | 9 | Guerra and Leite 2017 |
| Periurban | RBT | Reserva Biológica do Tinguá – Nova Iguaçu/ Duque de Caxias/ Petrópolis/ Miguel Pereira | 6 | Travassos et al. 2018 |
| Periurban | PEFI | Parque Estadual das Fontes do Ipiranga – São Paulo | 5 | Monticelli et al. 2021 |
| Preserved area | PEI | Parque Estadual Intervales – São Paulo | 18 | Vieira and Monteiro-Filho 2003 |
| Preserved area | RebioPA | Reserva Biológica Poço das Antas – Silva Jardin | 12 | Viveiros de Castro and Fernandes 2003 |
| Preserved area | PNI | Parque Nacional do Itatiaia - Itatiaia | 28 | Geise et al. 2004 |
| Preserved area | AGB | Aldeia Guarani do Bracuí – Angra dos Reis | 10 | Cunha and Rajão 2007 |
| Preserved area | PARNASO | Parque Nacional da Serra dos Órgãos – Teresópolis/ Petrópolis | 30 | Cronemberger et al. 2019 |
| Preserved area | PNSB | Parque Nacional da Serra da Bocaina - Paraty | 23 | Delciellos et al. 2023 |
